# Safety and Efficacy of Second-Generation Drug-Eluting Stents in Real-World Practice: Insights from the Multicenter Grand-DES Registry

**DOI:** 10.1155/2020/3872704

**Published:** 2020-02-26

**Authors:** You-Jeong Ki, Kyung Woo Park, Jeehoon Kang, Chee-Hoon Kim, Jung-Kyu Han, Han-Mo Yang, Hyun-Jae Kang, Bon-Kwon Koo, Hyo-Soo Kim

**Affiliations:** Department of Internal Medicine and Cardiovascular Center, Seoul National University Hospital, Seoul 03080, Republic of Korea

## Abstract

**Objective:**

In this study, we sought to compare the efficacy and safety of the Xience Prime/Xience V/Promus EES and Biomatrix/Biomatrix Flex/Nobori BES with resolute integrity/resolute ZES using the grand drug-eluting stent (Grand-DES) registry.

**Background:**

Currently, new-generation drug-eluting stents (DESs) are used as the standard of care in patients undergoing percutaneous coronary intervention. No study has simultaneously compared everolimus-eluting stent (EES), biolimus-eluting stent (BES), and zotarolimus-eluting stent (ZES).

**Methods:**

Stent-related composite outcomes (target lesion failure) and patient-related composite outcomes were compared in crude and propensity score-matched analysis.

**Results:**

Of the 17,286 patients in the Grand-DES group, 5,137, 2,970, and 4,990 patients in the EES, BES, and ZES groups completed a three-year follow-up. In the propensity score-matched cohort, the stent-related outcome (EES vs. BES vs. ZES; 5.9% vs. 6.7% vs. 7.1%, *P* = 0.226) and patient-related outcomes (12.7% vs. 13.5% vs. 14.3%, *P* = 0.226) and patient-related outcomes (12.7% vs. 13.5% vs. 14.3%, *P* = 0.226) and patient-related outcomes (12.7% vs. 13.5% vs. 14.3%, *P* = 0.226) and patient-related outcomes (12.7% vs. 13.5% vs. 14.3%,

**Conclusions:**

In this robust real-world registry with unrestricted use of EES, BES, and ZES, the three stent groups showed comparable safety and efficacy at the 3-year follow-up.

## 1. Introduction

Although the restenosis rate of bare metal stent is high, restenosis or stent thrombosis (ST) is known to be low after one year of revascularization [1]. Second-generation drug-eluting stents (DESs) were developed to improve the long-term efficacy and safety of patients receiving percutaneous coronary intervention (PCI), as first-generation DESs have been reported to have increased risk of late and very late ST and delayed catch-up and neoatherosclerosis [1, 2]. There are multiple studies reporting the short-term outcome within two years of real-world use of second-generation DESs, but there is significantly less data on the long-term outcomes from real-world use [3–5]. Although long-term data are comparable for everolimus-eluting stent (EES) and zotarolimus-eluting resolute stent (ZES) in several studies, there are only a few studies in this regard [3, 4, 6]. In the TWENTE II trial, the 5-year clinical outcome was similar between EES and ZES [7].

It remains to be seen whether the outcomes among different types of second-generation DESs are different including those that have biodegradable and biocompatible durable polymers. There are only a few reports on comparison of short-term data of biodegradable polymer and durable polymer-coated stents [8]. Furthermore, previous trials are limited to evaluating low-frequency adverse events, in particular very late ST, and long-term data are limited [4, 9, 10].

In the present study, we obtained the long-term 3-year clinical outcomes of second-generation DESs from the grand drug-eluting stent (Grand-DES) registry, a composite registry of a series of multicenter registries that include data of over 17,000 patients and compared individual DES groups. Detailed analysis of ST and its predictors are also presented.

## 2. Materials and Methods

### 2.1. Study Design and Patient Population

**Figure 1:**
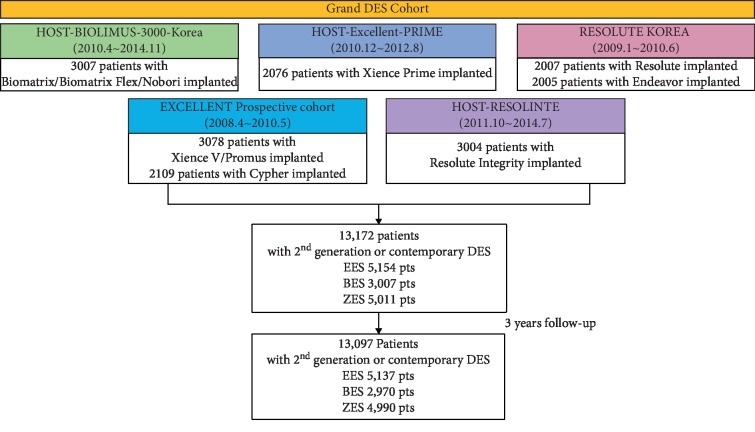
Flow diagram of participants. Grand-DES registry includes 5 multicenter registries—HOST-biolimus-3000-Korea, HOST-Excellent-Prime, EXCELLENT prospective cohort, HOST-Resolinte, and Resolute-Korea—that enrolled all-comers treated with ≥1 DES. DES = drug-eluting stent; EES = everolimus-eluting stent; BES = biolimus-eluting stent; ZES = zotarolimus-eluting stent.

This study evaluated the clinical outcomes of the EES, biolimus-eluting stent (BES), and ZES from Grand-DES registry. Grand-DES registry includes 5 multicenter registries—HOST-biolimus-3000-Korea, HOST-Excellent-Prime, EXCELLENT prospective cohort, HOST-Resolinte and Resolute-Korea—that enrolled all-comers treated with ≥1 DES without exclusions (Figure 1). The final sample size of the Grand-DES registry was 17,286 patients from 55 centers in Korea from January 1, 2004, to November 31, 2014. Among these patients, 13,172 patients were treated with new-generation DES. The new-generation stent used in this trial includes EES (Xience Prime/Xience V/Promus) with durable polymer, BES (Biomatrix/Biomatrix Flex/Nobori) with biodegradable polymer, and RES (resolute/resolute integrity) with durable polymer. The study complied with the provisions of the Declaration of Helsinki, and the study was approved by the institutional review board at each center. All patients provided written informed consent.

### 2.2. Procedure and Data Collection

All consecutive patients undergoing coronary angiography and PCI were included. Angioplasty and stenting were performed according to standard protocols chosen by cardiologist. The choice of the stent, predilatation, poststenting adjunctive balloon inflation, and the use of intravascular ultrasound or glycoprotein IIb/IIIa inhibitors were all left to the operators' discretion. All patients were prescribed aspirin daily 100 mg indefinitely and clopidogrel daily 75 mg for 1 year, after a loading dose of 300 mg or 600 mg. After index PCI, follow-ups were performed at 1, 3, 9, and 12 months and annually thereafter; repeat angiography was optional at 9 to 13 months. Clinical follow-up data were obtained from outpatient visits or by telephone call and/or medical questionnaire. For any clinical events, all relevant medical records were reviewed and adjudicated by an external clinical event committee. With the use of the Korean health system's unique identification numbers, the vital status of 100% of patients was cross checked. The median follow-up duration was 1,126 days (interquartile range 1,102-1,142 days).

### 2.3. End Points and Definitions

The primary outcome was target lesion failure (TLF), a composite of cardiac death, myocardial infarction (MI) (not clearly attributed to a nontarget vessel), or target lesion revascularization (TLR). Secondary outcome, patient-oriented composite outcome (POCO), included all-cause death, any MI, and any revascularization. Other secondary outcomes included individual elements of TLF and POCO, and ST, defined as probable and definite. TLR is considered clinically indicated if angiography at follow-up shows a diameter stenosis ≥50% and if one of following occurs: (1) A positive history of recurrent angina pectoris, presumably related to the target vessel, (2) objective signs of ischemia at rest (electrocardiogram changes) or during exercise test (or equivalent), presumably related to the target vessel, (3) abnormal results of any invasive functional diagnostic test, and (4) a TLR of target vessel revascularization (TVR) with a diameter stenosis ≥70% even in the absence of the abovementioned ischemic signs or symptoms. The definition of definite or probable ST is based on criteria provided by the Academic Research Consortium [11]. Bleeding events were defined by thrombolysis in myocardial infarction criteria [12].

### 2.4. Statistical Analysis

Categorical and continuous variables are given as counts (percentages) and mean ± standard deviation. To compare three stent groups, we used the *χ*2 test for categorical variables. And the mean values between the three groups were compared by analysis of variance followed by Tukey's honest significant difference test among different groups.

Time-dependent event occurrence rate was estimated by the Kaplan–Meier method with log-rank tests and Cox proportional hazard model. If the combined end points were occurred in one patient, the first event was counted. To compensate for the nonrandomized design of observational study, we used propensity score methods generated by a logistic regression model. Covariates for this matching model were selected if they differed significantly among the three groups or were clinically important. The covariates included binary variables (gender, diabetes mellitus, hypertension, dyslipidemia, chronic renal failure [CRF], previous MI, ST-segment elevation myocardial infarction [STEMI], non-ST-segment elevation myocardial infarction [NSTEMI], American College of Cardiology/American Heart Association B2/C lesions, long lesion [≥28 mm], small diameter [≤2.75 mm]) and continuous variable (age).

The effect of the variables on ST was evaluated using the univariate Cox proportional hazards models. Variables with *P*-value <0.05 in the univariate analysis were included in multivariable Cox proportional hazard analysis. Results are reported as hazard ratios (HRs) with 95% confidence intervals (CI). All statistical analyses were conducted with SPSS V.22 (IBM SPSS Statistics, Chicago, Illinois, USA) and R V. 3.4.1 (R Foundation for Statistical Computing, Vienna, Austria). A two-sided *P*-value <0.05 was considered as significant.

## 3. Results

### 3.1. Baseline Characteristics

The baseline characteristics of the study subjects are listed in Table 1. Of the 17,286 patients in the Grand-DES cohorts, 13,172 (76.2%) patients were treated with second-generation DESs, and the remaining 4,114 (23.8%) patients were treated with first-generation DESs. In the second-generation DES group, 5,154 (39.1%), 3,007 (22.8%), and 5,011 (38.0%) patients were treated with the EES, BES, and ZES, respectively. The mean age was 64.0 ± 10.9 years, and 70% were men. Furthermore, 29.4% of patients underwent PCI for MI and one-third for unstable angina. The proportion of patients diagnosed with STEMI was 14.7%. A total of 46.1% of the patients were revascularized for small diameter vessel and 29.8% for long lesion. A mean of 1.6 ± 0.9 stents were used per person. Patients treated with EES had a higher rate of diabetes mellitus and history of MI. Patients presented with STEMI were more frequent in the BES group. The rate of left main disease or multiple lesions was similar among the three groups. The American College of Cardiology/American Heart Association B2/C lesions were lower in the EES group (75.8%) than in the BES (79.6%) and ZES groups (79.8%). Procedural success rate was similar among the three groups. The rate of angiography follow-up was higher in the BES group (46.6%) than in the EES (37.9%) and ZES groups (39.6%) (*P* < 0.001). The number of protocol violations was 14 in the EES group, 11 in the BES group, and 15 in the ZES group, which were all a patients' withdrawal of consent. Furthermore, 3, 26, and 6 patients were lost to follow-up in the EES, BES, and ZES groups, respectively. Complete 3-year follow-up was achieved in 99.4% of patients. Finally, the number of patients used in the 3-year outcome analysis was 5,137, 2,970, and 4,990 in the EES, BES, and ZES groups, respectively. Other procedural and angiographic characteristics are described in Table 1.

### 3.2. Clinical Outcomes of EES, BES, and ZES Groups

With the use of second-generation stents, the cumulative incidence of cardiac events was 6.8% for TLF, 14.2% for POCO, and 10.8% for major adverse cardiac event (MACE). At 1 year, the cumulative incidence of TLF was 3.8%, POCO was 7.9%, and MACE was 5.0%.

**Figure 2:**
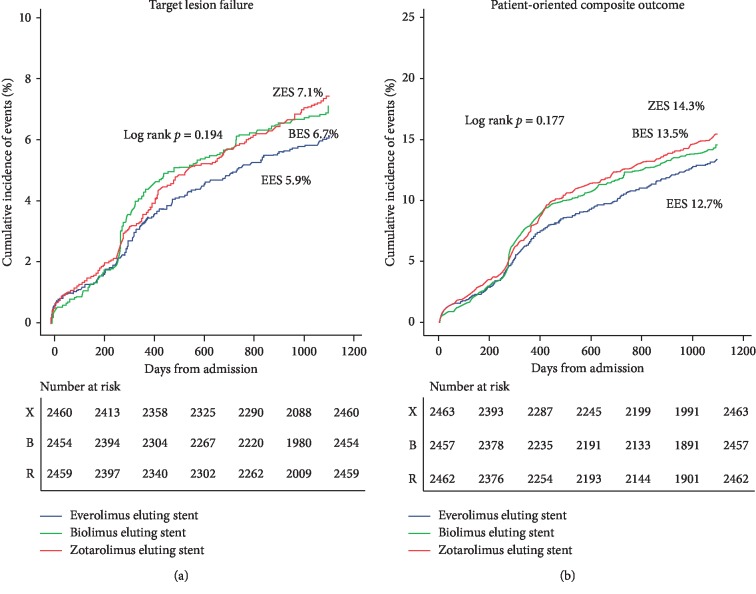
Survival analysis. Primary and major secondary outcomes in matched population. EES = everolimus-eluting stent; BES = biolimus-eluting stent; ZES = zotarolimus-eluting stent.

When matched with covariates, the event rate of TLF was 5.9% in the EES group, 6.7% in the BES group, and 7.1% in the ZES group (*P* = 0.226) (Table 2, Supplementary Table 1). When individual components of the primary end point were examined, the rate of cardiac death or MI was similar. Compared with that of EES, BES was associated with a higher rate of TLR (BES vs. EES, 3.9% vs. 2.8%; HR 1.419; 95% CI 1.040–1.938; *P* = 0.027) but did not result in difference in the TLF. There were no significant differences in the rate of all-cause death, cardiac cause of death, any MI, target vessel MI, any revascularization, ST, and major bleeding (Table 2). The comparison of clinical outcomes in the propensity score-matched population showed similar results to the Cox proportional hazard model. The cumulative incidence of TLF and POCO did not differ among the three stent groups (Figure 2). Although the incidence of TLF in the BES group was higher than that in the other stent groups during 1–2 years after PCI, the delayed catch-up was observed at 3-year follow-up. The EES group showed lower incidence of TLF and POCO than that of the BES and ZES groups.

**Figure 3:**
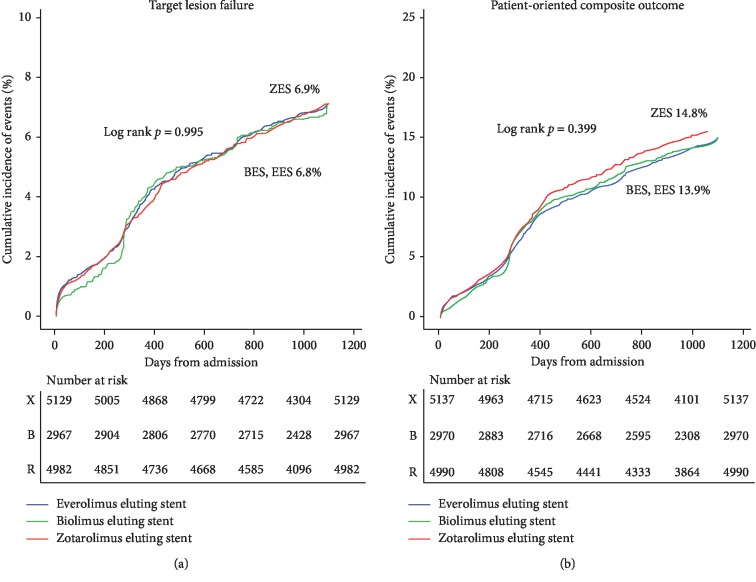
Survival analysis. Primary and major secondary outcomes in crude population. EES = everolimus-eluting stent; BES = biolimus-eluting stent; ZES = zotarolimus-eluting stent.

The crude population showed similar results with the propensity score-matched population. The cumulative incidence of TLF (death from cardiac causes, target vessel MI, or TLR) and POCO did not differ among the three stent groups (Figure 3). By stent classification, the 3-year cumulative incidence of cardiac death was higher in patients with ZES (4.1%) than in patients with BES (3.2%) (BES vs. ZES; HR 0.782; 95% CI 0.613–0.997; *P*=0.048). The incidence of TLR was higher in patients with BES (3.8%) than in patients with ZES (2.8%) (BES vs. ZES; HR 1.354; 95% CI 1.058–1.734; *P*=0.016) but did not result in significant difference in TLF (death from cardiac causes, target vessel MI, or TLR). The event rate for the primary end point was 6.8% in the EES and BES groups and 6.9% in the ZES group at 3-year follow-up (*P*=0.997). There were no significant differences between the groups in the rate of all-cause death, any MI, target vessel MI, any revascularization, ST, and major bleeding (Table 3, Supplementary [Supplementary-material supplementary-material-1]).

**Figure 4:**
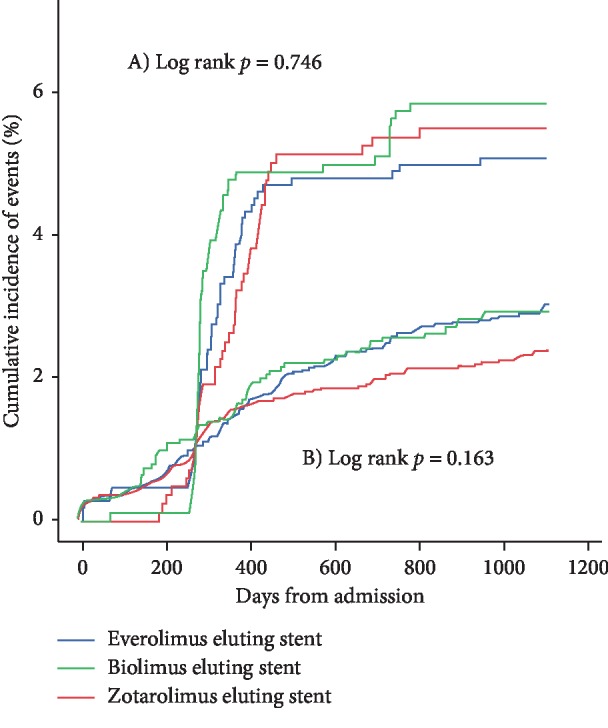
Survival curve of TLR. Differences in TLR divided by whether angiography was performed. (a) Group with dedicated angiography and (b) group without dedicated angiography. TLR = target lesion revascularization.

We compared the incidence of TLR according to the presence or absence of dedicated angiography follow-up. When divided them according to dedicated angiography, the difference in the rate of TLR disappeared (Figure 4).

### 3.3. Risk Factors for Stent Thrombosis

Definite or probable ST occurred in 85 (0.6%) patients at 3-year follow-up, 9 patients with acute ST (0 to 1 days), 42 patients with subacute ST (2 to 30 days), 18 patients with late ST (31 days to 1 year), and 16 patients with very late ST (>1 year) (Supplementary [Supplementary-material supplementary-material-1]). Furthermore, 60% of the ST occurred within the first 1 month after stent implantation. There was no significant difference in the ST occurrence rate among the three stent groups.  Table 4 shows the independent predictor of ST. Early ST (≤30 days after the index PCI) predictors were old age (≥65 years) and left ventricular dysfunction (ejection failure [EF] ≤ 40%). Late predictors of ST (>30 days) included CRF, previous PCI, left ventricular dysfunction, and American College of Cardiology/American Heart Association B2/C lesions. In all ST, multivariable Cox hazard analysis revealed that age ≥65 (HR 2.107; 95% CI 1.261–3.519; *P*=0.004), CRF (HR 3.178; 95% CI 1.621–6.229; *P*=0.001), previous PCI (HR 2.019; 95% CI 1.134–3.595; *P*=0.017), left ventricular dysfunction (HR 2.255; 95% CI 1.234–4.121; *P*=0.008), and premature single antiplatelet therapy (dual antiplatelet therapy less than 1 year) (HR 2.162; 95% CI 1.355–3.448; *P*=0.001) were the independent predictors of ST.

## 4. Discussion

To the best of our knowledge, this is the first and large observational study comparing EES, BES, and ZES simultaneously. A patient-level pooled analysis of 13,097 patients in the Grand-DES registry demonstrated the following. (1) The TLF and POCO were similar among the EES, BES, and ZES groups, respectively, in the 3-year outcome. (2) Despite a higher rate of TLR in the BES group, there was no significant difference among the three groups in the stent-related and patient-related outcomes in propensity score-matched populations. (3) The rates of definite and probable ST were comparable among the EES, BES, and ZES groups. (4) Old age (age ≥65), CRF, previous PCI, left ventricular dysfunction, and premature dual antiplatelet therapy discontinuation were the independent predictors of ST. The type of stent used itself was not a predictor of ST.

The patients in this registry had different baseline characteristics and angiographic characteristics, which is an inevitable feature of registry data. This limitation was overcome through propensity score matching. The results of propensity score-matched analysis were similar to those of crude population analysis.

The first-generation stent, which is known to reduce the rate of restenosis [5], is also known to have a risk of very late (>1 year) ST. The remaining polymer has been known to cause a sustained inflammatory response and inhibits endothelialization to enhance ST [1, 2]. These results have led to the development of next-generation stents. The second-generation DESs enhance biocompatibility of the polymers and incorporate a thinner cobalt-chromium stent platform to enhance endothelial coverage of stent struts and reduce ST risk. Currently, the most frequently used second-generation DESs, namely EES and ZES, showed similar safety and efficacy profiles [3, 4]. In EES and ZES, polymers are applied to the stent surface, indefinitely controlling drug delivery. To overcome the limitation of polymers, biodegradable polymer stents were developed. This stent is coated only at the abluminal site with a biodegradable polymer layer (20 mcg) that dissolves 6–9 months after implantation and from which the lipophilic antiproliferative drug biolimus elutes. Previous studies have demonstrated that BESs are as effective and safe as EES or ZES [8, 13–15].

This study is the first to compare biodegradable polymer BES and durable polymer EES and ZES simultaneously in large population. The results demonstrated that biodegradable polymer BES is as efficacious and safe as EES and ZES for up to 36 months after PCI, but with an apparent higher rate of TLR in the BES group. In the propensity score-matched cohort, the TLR rates were higher in the BES group than in the EES and ZES groups. In large patient-level pooled analysis of the NEXT and COMPARE II randomized trials, BES and EES resulted in similar outcomes but with a higher rate of target vessel MI in the BES group [13, 14]. These results might be because the BES is thicker than other stents. Indeed, thick strut stent is associated with significant restenosis after coronary artery stenting [16]. Although the biodegradable polymer DES is noninferior to the next-generation durable polymer DES in terms of safety and efficacy, continuous research is needed to ensure that it remains stable even after polymer dissolution.

Stent thrombosis is a serious adverse event commonly associated with cardiac death or acute MI. In fact, in our study, 38.8% of the ST events were fatal. Even among patients with very late ST, 12 of 16 patients eventually died or had MI. The definite or probable ST incidences up to 1 year in this study were good for EES (0.6%), BES (0.5%), and ZES (0.5%), compared with those reported previously [7, 13]. In a study of the final 5-year report of the LEADERS trial, the definite/probable ST at 1 year was 2.7% in the BES group and 2.2% in the sirolimus-eluting stent group. The incidence of ST during 1–5 years was lower in the BES group than in the sirolimus-eluting stent group (RR 0.26; 95% CI 0.10–0.68; *P* = 0.003) [17].

The mechanism of early and late ST is known to be different [18]. The occurrence of early ST is associated with postinterventional platelet aggregation [19] or procedure problem [20]. The occurrence of late ST is related to delayed healing and impaired endothelialization induced by drug polymer [20]. Our study also confirmed that the risk factors of early and late ST were different. The occurrence of late ST was related to delayed healing and impaired endothelialization induced by drug polymer [1, 20]. Theoretically, the rate of ST was expected to be low in the BES group because the polymer disappeared after 6–9 months. However, in our study, the type of stent itself was not a predictor of ST. Other studies have demonstrated that patients with renal failure, undergoing treatment for in-stent restenosis and bifurcation lesions, premature antiplatelet therapy discontinuation, and left ventricular dysfunction were associated with late ST [9, 10, 21].

The advantage of this study is that it is a large sample size study comparing the second-generation EES, BES, and ZES simultaneously. We obtained the long-term 3-year clinical outcomes of second-generation DES. Furthermore, detailed analysis of ST and its predictors is also evaluated.

## 5. Limitations

Our study had some limitations. Because of the retrospective nonrandomized nature of analysis, stent selection bias must be assumed. Uneven distribution of risk factors should be considered. Although risk factor adjustment through propensity score matching was conducted, unmeasured variables could not be matched. The incidence of MI was relatively lower than that in other studies. Because postprocedural collection of cardiac markers to detect MI was not routinely performed, the true rate of MI might be higher than described.

## 6. Conclusions

In this robust real-world registry with unrestricted use of EES, BES, and ZES, the three stent groups showed comparable safety and efficacy at 3-year follow-up, apart from TLR, which occurred more frequently in the BES group than in the EES or ZES group in the matched population. In the multivariate analysis, chronic kidney disease was the strongest predictor of stent thrombosis. The type of stent used itself was not a predictor of ST.

## Figures and Tables

**Table 1:** Baseline characteristics.

	Total (*n* = 13,172)	EES (*n* = 5,154)	BES (*n* = 3,007)	ZES (*n* = 5,011)	*P* value
Demographics					
Age (years)	64.0 ± 10.9	64.0 ± 10.8	64.0 ± 11.0	64.1 ± 11.0	0.621
Male (*n*, %)	9216/13172 (70.0%)	3537/5154 (68.6%)	2119/3007 (70.5%)	3560/5011 (71.0%)	0.023
Body mass index (kg/m^2^)	24.6 ± 3.2	24.6 ± 3.2	24.6 ± 3.3	24.6 ± 3.1	0.911
Coexisting condition					
Diabetes mellitus (*n*, %)	4828/13172 (35.9%)	1912/5154 (37.1%)	1032/3007 (34.3%)	1784/5011 (35.6%)	0.036
Hypertension (*n*, %)	8109/13172 (61.6%)	3226/5154 (62.6%)	1766/3007 (58.7%)	3117/5011 (62.2%)	0.001
Dyslipidemia (*n*, %)	7966/13172 (60.5%)	2705/5154 (52.5%)	2080/3007 (69.2%)	3181/5011 (63.5%)	<0.001
Peripheral vascular disease (*n*, %)	259/13172 (2.0%)	90/5154 (1.7%)	58/3007 (1.9%)	111/5011 (2.2%)	0.231
Chronic renal failure (*n*, %)	572/13172 (4.3%)	217/5154 (4.2%)	121/3007 (4.0%)	234/5011 (4.7%)	0.326
Cardiac risk factors					
Current smoker (*n*, %)	3936/13172 (29.9%)	1573/5154 (30.5%)	890/3007 (29.6%)	1473/5011 (29.4%)	0.431
Previous myocardial infarction (*n*, %)	739/13172 (5.6%)	346/5154 (6.7%)	146/3007 (4.9%)	247/5011 (4.9%)	<0.001
Previous PCI (*n*, %)	1941/13172 (14.7%)	762/5154 (14.8%)	401/3007 (13.3%)	778/5011 (15.5%)	0.027
Previous coronary artery bypass surgery (*n*, %)	239/13172 (1.8%)	95/5154 (1.8%)	56/3007 (1.9%)	88/5011 (1.8%)	0.924
Family history of coronary artery disease (*n*, %)	789/13172 (6.0%)	284/5154 (5.5%)	189/3007 (6.3%)	316/5011 (6.3%)	0.177
Left ventricular ejection fraction <40% (*n*, %)	746/11267 (6.6%)	318/4604 (6.9%)	137/2465 (5.6%)	291/4198 (6.9%)	0.056
Clinical indication of PCI					
STEMI (*n*, %)	1926/13064 (14.7%)	717/5122 (14.0%)	484/2967 (16.3%)	725/4975 (14.6%)	0.017
NSTEMI (*n*, %)	1922/13064 (14.7%)	728/5122 (14.2%)	463/2967 (15.6%)	731/4975 (14.7%)	0.234
Unstable angina (*n*, %)	4385/13064 (33.6%)	1772/5122 (34.6%)	929/2967 (31.3%)	1684/4975 (33.8%)	0.009
Stable angina (*n*, %)	4317/13064 (33.0%)	1650/5122 (32.2%)	1006/2967 (33.9%)	1661/4975 (33.4%)	0.240
Silent ischemia (*n*, %)	514/13064 (3.9%)	255/5122 (5.0%)	85/2967 (2.9%)	174/4975 (3.5%)	<0.001
Lesion characteristics					
Left main disease (*n*, %)	820/13172 (6.2%)	329/5154 (6.4%)	170/3007 (5.7%)	321/5011 (6.4%)	0.335
Multiple target lesions (*n*, %)	3872/13172 (29.4%)	1542/5154 (29.9%)	831/3007 (27.6%)	1499/5011 (29.9%)	0.055
At least on target lesion with					
Type B2/C lesion (*n*, %)	10301/13172 (78.2%)	3905/5154 (75.8%)	2395/30007 (79.6%)	4001/5011 (79.8%)	<0.001
Bifurcation (*n*, %)	5339/13172 (40.5%)	1918/5154 (37.2%)	1284/3007 (42.7%)	2137/5011 (42.6%)	<0.001
Severe calcification (n, %)	1151/13172 (8.7%)	437/5154 (8.5%)	311/3007 (10.3%)	403/5011 (8.0%)	0.001
Tortuosity (>45°) (*n*, %)	2821/13172 (21.4%)	1140/5154 (22.1%)	600/3007 (20.0%)	1081/5011 (21.6%)	0.067
Thrombotic total (*n*, %)	1438/13172 (10.9%)	505/5154 (9.8%)	375/3007 (12.5%)	558/5011 (11.1%)	0.001
ISR as a target lesion (*n*, %)	505/13151 (3.8%)	225/5139 (4.4%)	105/3007 (3.5%)	175/5005 (3.5%)	0.037
Long lesion (≥28 mm) (*n*, %)	3925/13172 (29.8%)	1756/5154 (34.1%)	547/3007 (18.2%)	1622/5011 (32.4%)	<0.001
Small diameter (≤2.75 mm) (*n*, %)	6066/13172 (46.1%)	2473/5154 (48.0%)	1236/3007 (41.1%)	2357/5011 (47.0 %)	<0.001
IVUS-guided (*n*, %)	4946/13172 (37.5%)	2033/5154 (39.4%)	1022/3007 (34.0%)	1891/5011 (37.7%)	<0.001
Previous treated lesion (*n*, %)	984/13172 (7.5%)	404/5154 (7.8%)	151/3007 (5.0%)	429/5011 (8.6%)	<0.001
Side branch treatment (*n*, %)	1176/13172 (8.9%)	408/5154 (7.9%)	320/3007 (10.6%)	448/5011 (8.9%)	<0.001
Number of stents	1.6 ± 0.9	1.7 ± 0.9	1.6 ± 0.9	1.6 ± 0.9	<0.001
Stent average diameter (mm)	3.1 ± 0.5	3.1 ± 0.4	3.1 ± 0.4	3.0 ± 0.5	0.001
Total stent length (mm)	38.8 ± 25.7	39.8 ± 26.1	34.8 ± 23.4	40.3 ± 26.4	<0.001
Device success (*n*, %)	12949/13170 (98.3%)	5074/5152 (98.5%)	2947/3007 (98.0%)	4928/5011 (98.3%)	0.261
Lesion success (*n*, %)	12927/13170 (98.2%)	5069/5152 (98.4%)	2944/3007 (97.9%)	4914/5011 (98.1%)	0.244
Procedural success (*n*, %)	12914/13170 (98.1%)	5061/5152 (98.2%)	2941/3007 (97.8%)	4912/5011 (98.0%)	0.392
Angiography f/*u* (*n*, %)	5340/13172 (40.5%)	1954/5152 (37.9%)	1401/3007 (46.6%)	1985/5011 (39.6%)	<0.001
Dedicated angiography f/*u* (*n*, %)	2866/13172 (21.8%)	1085/5152 (21.1%)	943/3007 (31.4%)	838/5011 (16.7%)	<0.001
DAPT duration (days)	689 ± 373	711 ± 375	674 ± 374	675 ± 371	<0.001

Data are mean (±SD). EES = everolimus-eluting stent; BES = biolimus-eluting stent; ZES = zotarolimus-eluting stent; PCI = percutaneous coronary intervention; STEMI = ST-segment elevation myocardial infarction; NSTEMI = non-ST-segment elevation myocardial infarction; ISR = in-stent restenosis; IVUS = intravascular ultrasound; DAPT = dual antiplatelet therapy.

**Table 2:** Clinical outcomes in matched population at 3-year follow-up.

	Total (*n* = 7,389)	EES (*n* = 2,463)	BES (*n* = 2,463)	ZES (*n* = 2,463)	*P* value
Target lesion failure^*∗*^	485 (6.6%)	145 (5.9%)	166 (6.7%)	174 (7.1%)	0.226
POCO^†^	997 (13.5%)	312 (12.7%)	332 (13.5%)	353 (14.3%)	0.232
All-cause death	431 (5.8%)	135 (5.5%)	141 (5.7%)	155 (6.3%)	0.459
Cardiac death	255 (3.5%)	78 (3.2%)	78 (3.2%)	99 (4.0%)	0.167
Myocardial infarction	97 (1.3%)	29 (1.2%)	33 (1.3%)	35 (1.4%)	0.746
Target vessel myocardial infarction	50 (0.7%)	11 (0.4%)	21 (0.9%)	18 (0.7%)	0.204
Any revascularization	585 (7.9%)	180 (7.3%)	203 (8.2%)	202 (8.2%)	0.390
Target lesion revascularization	238 (3.2%)	68 (2.8%)	95 (3.9%)	75 (3.0%)	0.078
Stent thrombosis^‡^	47 (0.6%)	15 (0.6%)	19 (0.8%)	13 (0.5%)	0.549
Major bleeding	78 (1.1%)	28 (1.1%)	22 (0.9%)	28 (1.1%)	0.627

Values are *n* (%), unless otherwise indicated. ^*∗*^Target lesion failure defined as a composite of cardiac death, MI (not clearly attributed to a nontarget vessel), or target lesion revascularization. ^†^POCO includes all-cause mortality, any MI (includes nontarget vessel territory), and revascularization. ^‡^Stent thrombosis includes definite and probable stent thrombosis. EES = everolimus-eluting stent; BES = biolimus-eluting stent; ZES = zotarolimus-eluting stent; POCO = patient-oriented composite outcome; MI = myocardial infarction.

**Table 3:** Clinical outcomes in crude population at 3-year follow-up.

	Total (*n* = 13,097)	EES (*n* = 5,137)	BES (*n* = 2,970)	ZES (*n* = 4,990)	*P* value
Target lesion failure^*∗*^	895 (6.8%)	350 (6.8%)	203 (6.8%)	342 (6.9%)	0.997
POCO^†^	1862 (14.2%)	712 (13.9%)	413 (13.9%)	737 (14.8%)	0.364
All-cause death	815 (6.2%)	316 (6.2%)	168 (5.7%)	331 (6.6%)	0.210
Cardiac death	481 (3.7%)	183 (3.6%)	95 (3.2%)	203 (4.1%)	0.118
Myocardial infarction	158 (1.2%)	58 (1.1%)	44 (1.5%)	56 (1.1%)	0.295
Target vessel myocardial infarction	84 (0.6%)	29 (0.6%)	26 (0.9%)	29 (0.6%)	0.191
Any revascularization	1075 (8.2%)	406 (7.9%)	258 (8.7%)	411 (8.2%)	0.463
Target lesion revascularization	428 (3.3%)	173 (3.4%)	114 (3.8%)	141 (2.8%)	0.043
Stent thrombosis^‡^	85 (0.6%)	34 (0.7%)	22 (0.7%)	29 (0.6%)	0.685
Major bleeding	167 (1.3%)	77 (1.5%)	27 (0.9%)	63 (1.3%)	0.074

Values are *n* (%), unless otherwise indicated. ^*∗*^Target lesion failure defined as a composite of cardiac death, MI (not clearly attributed to a nontarget vessel), or target lesion revascularization. ^†^POCO includes all-cause mortality, any MI (includes nontarget vessel territory), and revascularization. ^‡^Stent thrombosis includes definite and probable stent thrombosis. EES = everolimus-eluting stent; BES = biolimus-eluting stent; ZES = zotarolimus-eluting stent; POCO = patient-oriented composite outcome; MI = myocardial infarction.

**Table 4:** Independent predictors of stent thrombosis in crude population group.^*∗*^

	HR (95% CI)	*P* value
Cumulative stent thrombosis^†^		
Age ≥65	2.107 (1.261–3.519)	0.004
Chronic renal failure	3.178 (1.621–6.229)	0.001
Previous PCI	2.019 (1.134–3.595)	0.017
Left ventricular ejection fraction <40%	2.255 (1.234–4.121)	0.008
Premature SAPT	2.162 (1.355–3.448)	0.001

Early stent thrombosis (0–30 days)^‡^		
Age ≥65	3.735 (1.715–8.133)	0.001
Left ventricular ejection fraction <40%	2.393 (1.070–5.355)	0.034

Late stent thrombosis (31 days)^§^		
Chronic renal failure	4.668 (1.881–11.581)	0.001
Previous PCI	3.967 (1.716–9.172)	0.001
Left ventricular ejection fraction <40%	2.514 (1.001–6.312)	0.050
Type B2/C	7.667 (1.027–57.224)	0.047

^∗^Identification of independent predictors was done with Cox proportional hazard regression model, and the variables were presented with multivariable-adjusted HRs, 95% CI, and *P*-values. ^†^Variables included in the model are old age, diabetes mellitus, chronic renal failure, previous PCI, previous CABG, left ventricular dysfunction, premature SAPT, STEMI, multiple lesion, American College of Cardiology/American Heart Association B2/C lesions, previous treated lesion, total stent length, and major classification of used stent. ^‡^Variables included in the model are old age, diabetes mellitus, chronic renal failure, previous MI, left ventricular dysfunction, STEMI, multiple lesion, American College of Cardiology/American Heart Association B2/C lesions, total stent length, small diameter, and major classification of used stent. ^§^Variables included in the model are old age, chronic renal failure, previous MI, previous PCI, left ventricular dysfunction, STEMI, premature SAPT, American College of Cardiology/American Heart Association B2/C lesions, previous treated lesion, long lesion, small diameter, and major classification of used stent. HR = hazard ratio; CI = confidence interval; PCI = percutaneous coronary intervention; SAPT = single antiplatelet agent therapy; STEMI = ST-segment elevation myocardial infarction; MI = myocardial infarction; CABG = coronary artery bypass surgery.

## Data Availability

The data used to support the findings of this study are available from the corresponding author upon request after the author gets approval of the ethics committee.

## Supplementary Materials

Supplementary Materials See Tables S1 and S2 in the Supplementary Material for comprehensive clinical outcomes in matched population and crude population group. See Table S3 in the Supplementary Material for comprehensive incidence and clinical presentation of stent thrombosis.
