# Supplementary material for: Safety and Efficacy of Second-Generation Drug-Eluting Stents in Real-World Practice: Insights from the Multicenter Grand-DES Registry
Source: J Interv Cardiol. 2020 Feb 26;2020:3872704. doi: 10.1155/2020/3872704 (PMC7061140; doi:10.1155/2020/3872704)
Supplement: Supplementary Materials — See Tables S1 and S2 in the Supplementary Material for comprehensive clinical outcomes in matched population and crude population group. See Table S3 in the Supplementary Material for comprehensive incidence and clinical presentation of stent thrombosis. [file 3872704.f1.docx]

Supplementary Table 1: Clinical outcomes in matched population at 3-year follow up

|  | Total  (n=7,389) | EES  (n=2,463) | BES  (n=2,463) | ZES  (n=2,463) | *P* value | BES vs. EES | | BES vs. ZES | | | ZES vs. EES | |  |
| --- | --- | --- | --- | --- | --- | --- | --- | --- | --- | --- | --- | --- | --- |
|  |  |  |  |  |  | **HR (95% CI)** | ***P* value** | **HR (95% CI)** | ***P* value** | **HR (95% CI)** | | ***P* value** | |
| Target lesion failure* | 485 (6.6%) | 145 (5.9%) | 166 (6.7%) | 174 (7.1%) | 0.226 | 1.165 (0.933-1.456) | 0.178 | 0.960 (0.776-1.188) | 0.710 | 1.213 (0.973-1.513) | | 0.085 | |
| POCO† | 997 (13.5%) | 312 (12.7%) | 332 (13.5%) | 353 (14.3%) | 0.232 | 1.083 (0.928-1.264) | 0.312 | 0.942 (0.811-1.095) | 0.436 | 1.149 (0.987-1.338) | | 0.073 | |
| All cause death | 431 (5.8%) | 135 (5.5%) | 141 (5.7%) | 155 (6.3%) | 0.459 | 1.059 (0.837-1.341) | 0.632 | 0.914 (0.727-1.148) | 0.438 | 1.159 (0.921-1.460) | | 0.209 | |
| Cardiac death | 255 (3.5%) | 78 (3.2%) | 78 (3.2%) | 99 (4.0%) | 0.167 | 1.014 (0.741-1.388) | 0.929 | 0.792 (0.588-1.065) | 0.122 | 1.282 (0.953-1.724) | | 0.101 | |
| MI | 97 (1.3%) | 29 (1.2%) | 33 (1.3%) | 35 (1.4%) | 0.746 | 1.156 (0.702-1.903) | 0.570 | 0.942 (0.586-1.516) | 0.807 | 1.226 (0.75-2.006) | | 0.416 | |
| Target vessel MI | 50 (0.7%) | 11 (0.4%) | 21 (0.9%) | 18 (0.7%) | 0.204 | 1.935 (0.933-4.014) | 0.076 | 1.164 (0.620-2.185) | 0.637 | 1.663 (0.785-3.521) | | 0.184 | |
| Any revascularization | 585 (7.9%) | 180 (7.3%) | 203 (8.2%) | 202 (8.2%) | 0.390 | 1.148 (0.939-1.403) | 0.179 | 1.009 (0.83-1.226) | 0.927 | 1.137 (0.930-1.390) | | 0.209 | |
| Target lesion revascularization | 238 (3.2%) | 68 (2.8%) | 95 (3.9%) | 75 (3.0%) | 0.078 | 1.419 (1.040-1.938) | 0.027 | 1.275 (0.942-1.726) | 0.116 | 1.113 (0.802-1.545) | | 0.522 | |
| Stent thrombosis^‡^ | 47 (0.6%) | 15 (0.6%) | 19 (0.8%) | 13 (0.5%) | 0.549 | 1.277 (0.649-2.514) | 0.478 | 1.467 (0.725-2.970) | 0.287 | 0.871 (0.414-1.830) | | 0.715 | |
| Major bleeding | 78 (1.1%) | 28 (1.1%) | 22 (0.9%) | 28 (1.1%) | 0.627 | 0.791 (0.452-1.382) | 0.410 | 0.774 (0.442-1.352) | 0.368 | 1.022 (0.605-1.726) | | 0.935 | |

Values are n (%), unless otherwise indicated.

^*^Target lesion failure defined as a composite of cardiac death, MI (not clearly attributed to a non-target vessel), or target lesion revascularization.

^†^ POCO includes all-cause mortality, any MI (includes non-target vessel territory), and revascularization.

^‡^Stent thrombosis includes definite and probable stent thrombosis.

EES, everolimus eluting stent; BES, biolimus eluting stent; ZES, zotarolimus eluting stent; HR, hazard ratio; CI, confidence interval; POCO, patient-oriented composite outcome; MI, myocardial infarction.

Supplementary Table 2: Clinical outcomes in crude population at 3-year follow up

|  | Total  (n=13,097) | EES  (n=5,137) | BES  (n=2,970) | ZES  (n=4,990) | *P* value | BES vs. EES | | BES vs. ZES | | | ZES vs. EES | | |  |
| --- | --- | --- | --- | --- | --- | --- | --- | --- | --- | --- | --- | --- | --- | --- |
|  |  |  |  |  |  | **HR (95% CI)** | ***P* value** | | **HR (95% CI)** | ***P* value** | | **HR (95% CI)** | ***P* value** | |
| Target lesion failure^*^ | 895 (6.8%) | 350 (6.8%) | 203 (6.8%) | 342 (6.9%) | 0.997 | 1.005 (0.846-1.195) | 0.952 | | 0.995 (0.836-1.184) | 0.955 | | 1.010 (0.870-1.173) | 0.893 | |
| POCO^†^ | 1862 (14.2%) | 712 (13.9%) | 413 (13.9%) | 737 (14.8%) | 0.364 | 1.006 (0.891-1.135) | 0.925 | | 0.935 (0.829-1.054) | 0.272 | | 1.076 (0.971-1.193) | 0.163 | |
| All cause death | 815 (6.2%) | 316 (6.2%) | 168 (5.7%) | 331 (6.6%) | 0.210 | 0.920 (0.763-1.109) | 0.383 | | 0.848 (0.704-1.020) | 0.081 | | 1.086 (0.930-1.266) | 0.297 | |
| Cardiac death | 481 (3.7%) | 183 (3.6%) | 95 (3.2%) | 203 (4.1%) | 0.118 | 0.899 (0.702-1.152) | 0.402 | | 0.782 (0.613-0.997) | 0.048 | | 1.151 (0.942-1.405) | 0.169 | |
| MI | 158 (1.2%) | 58 (1.1%) | 44 (1.5%) | 56 (1.1%) | 0.295 | 1.314 (0.888-1.944) | 0.172 | | 1.309 (0.882-1.943) | 0.181 | | 1.004 (0.695-1.449) | 0.985 | |
| Target vessel MI | 84 (0.6%) | 29 (0.6%) | 26 (0.9%) | 29 (0.6%) | 0.191 | 1.551 (0.913-2.633) | 0.104 | | 1.490 (0.878-2.530) | 0.140 | | 1.041 (0.622-1.741) | 0.879 | |
| Any revascularization | 1075 (8.2%) | 406 (7.9%) | 258 (8.7%) | 411 (8.2%) | 0.463 | 1.101 (0.942-1.287) | 0.227 | | 1.048 (0.897-1.225) | 0.551 | | 1.050 (0.916-1.204) | 0.485 | |
| Target lesion revascularization | 428 (3.3%) | 173 (3.4%) | 114 (3.8%) | 141 (2.8%) | 0.043 | 1.140 (0.900-1.444) | 0.278 | | 1.354 (1.058-1.734) | 0.016 | | 0.842 (0.674-1.051) | 0.129 | |
| Stent thrombosis^‡^ | 85 (0.6%) | 34 (0.7%) | 22 (0.7%) | 29 (0.6%) | 0.685 | 1.123 (0.657-1.920) | 0.672 | | 1.268 (0.729-2.208) | 0.400 | | 0.885 (0.539-1.453) | 0.630 | |
| Major bleeding | 167 (1.3%) | 77 (1.5%) | 27 (0.9%) | 63 (1.3%) | 0.074 | 0.603 (0.389-0.934) | 0.024 | | 0.702 (0.447-1.103) | 0.125 | | 0.858 (0.615-1.197) | 0.367 | |

Values are n (%), unless otherwise indicated.

^*^Target lesion failure defined as a composite of cardiac death, MI (not clearly attributed to a non-target vessel), or target lesion revascularization.

^†^ POCO includes all-cause mortality, any MI (includes non-target vessel territory), and revascularization.

^‡^Stent thrombosis includes definite and probable stent thrombosis.

EES, everolimus eluting stent; BES, biolimus eluting stent; ZES, zotarolimus eluting stent; HR, hazard ratio; CI, confidence interval; POCO, patient-oriented composite outcome; MI, myocardial infarction.

Supplementary Table 3: Stent thrombosis in crude population at 3 year

|  | Total  (n=13,097) | EES  (n=5,137) | BES  (n=2,970) | ZES  (n=4,990) | *P* value |
| --- | --- | --- | --- | --- | --- |
| ST (definite or probable) | 85 (0.6%) | 34 (0.7%) | 22 (0.7%) | 29 (0.6%) | 0.685 |
| Acute ST (0-1 day) | 9 (0.1%) | 5 (0.1%) | 1 (0.03%) | 3 (0.1%) | 0.549 |
| Subacute ST (2-30 days) | 42 (0.3%) | 18 (0.4%) | 13 (0.4%) | 11 (0.2%) | 0.225 |
| Late ST (31-365 days) | 18 (0.1%) | 6 (0.1%) | 2 (0.1%) | 10 (0.2%) | 0.264 |
| Very late ST (366- days) | 16 (0.1%) | 5 (0.1%) | 6 (0.2%) | 5 (0.1%) | 0.366 |
| Clinical presentation at time of ST |  |  |  |  | 0.579 |
| Cardiac death | 33/85 (38.8%) | 15/34 (44.1%) | 10/22 (45.5%) | 8/29 (27.6%) |  |
| Myocardial infarction | 36/85 (42.4%) | 12/34 (35.3%) | 9/22 (40.9%) | 15/29 (51.7%) |  |
| Revascularization | 16/85 (18.8%) | 7/34 (20.6%) | 3/22 (13.6%) | 6/29 (20.7%) |  |

EES, everolimus eluting stent; BES, biolimus eluting stent; ZES, zotarolimus eluting stent; ST, stent thrombosis.
